# Inhibitory effect of ethanol extract of *Nannochloropsis oceanica* on lipopolysaccharide-induced neuroinflammation, oxidative stress, amyloidogenesis and memory impairment

**DOI:** 10.18632/oncotarget.17268

**Published:** 2017-04-20

**Authors:** Ji Yeon Choi, Chul Ju Hwang, Hee Pom Lee, Hee Sik Kim, Sang-Bae Han, Jin Tae Hong

**Affiliations:** ^1^ College of Pharmacy and Medical Research Center, Chungbuk National University, Osong-eup, Heungdeok-gu, Cheongju, Chungbuk 28160, Republic of Korea; ^2^ Sustainable Bioresource Research Center, Korea Research Institute of Bioscience and Biotechnology (KRIBB), Yuseoung, Daejeon 305-806, Republic of Korea

**Keywords:** neuroinflammation, amyloidogenesis, oxidation, NF-κB, Nannochloropsis oceanica

## Abstract

Oxidative stress and neuroinflammation is implicated in the pathogenesis and development of Alzheimer's disease (AD). Here, we investigated the suppressive possibility of ethanol extract of *Nannochloropsis oceanica* (*N. oceanica*) on memory deficiency along with the fundamental mechanisms in lipopolysaccharide (LPS)-treated mice model. Among several extracts of 32 marine microalgae, ethanol extract of *N. oceanica* showed the most significant inhibitory effect on nitric oxide (NO) generation, NF-κB activity and β-secretase activity in cultured BV-2 cells, neuronal cells and Raw 264.7 cells. Ethanol extract of *N. oceanica* (50, 100 mg/kg) also ameliorated LPS (250 μg/kg)-induced memory impairment. We also found that ethanol extract of *N. oceanica* inhibited the LPS-induced expression of iNOS and COX-2. Furthermore, the production of reactive oxygen species (ROS), malondialdehyde (MDA) level as well as glutathione (GSH) level was also decreased by treatment of ethanol extract of *N.oceanica*. The ethanol extract of *N. oceanica* also suppresses IκB degradation as well as p50 and p65 translocation into the nucleus in LPS-treated mice brain. Associated with the inhibitory effect on neuroinflammation and oxidative stress, ethanol extract of *N. oceanica* suppressed Aβ_1-42_ generation through down-regulation of APP and BACE1 expression in *in vivo*. These results suggest that ethanol extract of *N. oceanica* ameliorated memory impairment via anti-inflammatory, anti-oxidant and anti-amyloidogenic mechanisms.

## INTRODUCTION

In recent years, algae have been the oldest living organisms on earth and considered a rich and sustainable source of bioactive compounds such as antioxidants, vitamins, carotenoids as well as fatty acids and amino acids and thus, utilized for biomass as food and feed additives [1, 2]. It is now a global trend to replace artificial antioxidants with natural sources since increased consumption in food supplements as bioactive compounds and in functional foods with safety issues [3, 4]. Microalgae could be used as alternative and renewable resource since they are a much more diverse biomass among the commercially available natural antioxidant sources [5]. The microalgae evaluation of antioxidant activity of some genera of *Chlorella* [6, 7], *Dunaliella* [8], *Spirulina* [9], *Botryococcus* [10], *Nostoc* [11], *Phaeodactylum* [12], *Halochlorococcum* [13], *Nannochloropsis* [14], and *Navicula* sp. [15] have been reported.

*Nannochloropsis sp*. including *oceanica* strain has been identified as well-known sources of eicosapentaenoic acids (EPA), docosahexaenoic acids (DHA), and etc, that are important polyunsaturated fatty acids [16]. These omega-3 unsaturated fatty acids have been reported to protect against generation of oxidative stress in neural cells [17] and against dementia including Alzheimer's disease (AD) [18]. EPA and DHA possess health-promoting effects and preventive activity of cancer, atherosclerosis, heart disease, arthritis and psoriasis [2, 19]. They are important in the brain and blood vessels and are considered to be vital for brain and retina development [2, 20]. There are a number of alternative EPA and DHA sources, including fungi, bacteria, plants, and microalgae that are already being investigated for commercial use [21]. However, algae are the most plentiful primary producers of EPA and DHA. It was also investigated that ethanol extract of *Nannochloropsis sp*. exhibits high antioxidant activity and when compared to water and ethanol/water extracts, ethanol extracts have higher activity than water extracts [14]. The ethanol/hexane extract of *Nannochloropsis* induced higher level of hepatic and plasma EPA and DHA and lowered the cholesterol levels in the rats which were fed hypercholesterolemic diets [22]. *Nannochlropsis sp*. also prevented As(III) induced reduction in growth variables and proliferation of some contaminant species [23]. It was also reported that *Nannochloropsis oceanica (N.oceanica)* has significantly promoted antioxidant activity in the amyloid beta-induced oxidative stress in neuronal cells and higher radical scavenging activity [21, 24]. However, to the extent of our knowledge, no work has been done about the biological effect of *Nannochloropsis sp*. on neuronal damages [25]. In the present study, the major fatty acid composition of ethanol extract of *N.oceanica* was determined by gas chromatography coupled to mass spectrometry (GC-MS). The extract contains about 44 types of fatty acids and one of the components was EPA/DHA. We expect EPA from microalgae based on their anti-oxidant and anti-inflammatory activities which could be a promising tool for the prevention of inflammatory diseases.

AD is an age-related neurodegenerative disease characterized by the accumulation of amyloid beta (Aβ), an insoluble peptide causing oxidative damages and neuroinflammation in the brain [26]. Brains of AD patients exhibit a number of pathological abnormalities including loss of synapses and glial function and inflammatory processes [27, 28]. Recently, we and other researchers have also shown that anti-inflammatory and anti-oxidative agents prevent Aβ deposition and brain damages, so these agents could be applicable for the preventive treatment in AD [29]. Furthermore, exposure of lipopolysaccharide (LPS) has been shown to have cognitive and long-term behavioral consequences [30, 31]. Together, these reports and our results indicate that acute induction of systemic inflammation causes brain inflammation. Although induced systemic inflammation model does not seem to be AD model, systemic LPS administration in mice can result in microglial activation and prolonged pro-inflammatory response that can enhance neuron damage and progressive neurodegeneration. However, a different strategy could be the bridge between AD and neuroinflammation since brain inflammation causes neurodegenerative diseases including AD.

Nuclear factor-kappa B (NF-κB) is a redox transcription factor that influences the levels of oxidative stress in cell [32, 33]. Expression of several inflammatory genes such as inducible nitric oxide synthase (iNOS) and cyclooxygenase-2 (COX-2) as well as inflammatory cytokines can be regulated by the activation of NF-κB [34]. Moreover, the promoter of neuronal BACE1, a limiting enzyme producing Aβ has NF-κB DNA consensus sequences [35]. Thus, blocking NF-κB could manage AD through the reduction of neuroinflammation, oxidative stress as well as amyloidogenesis [36]. Interestingly, epidemiologic studies have demonstrated that the anti-inflammatory and anti-oxidative therapies could decrease the risk of AD via reducing NF-κB activity [37].

In the present study, we investigated whether ethanol extract of *N. oceanica* has anti-amyloidogenic, anti-inflammatory as well as anti-oxidative properties, and thus ameliorates memory dysfunction in *in vivo* mice model.

## RESULTS

### Screening the inhibitory effects of extracts from marine microalgae on NO generation, NF- κB, β-secretase activities as well as cell viability

32 marine microalgae were extracted with several different solvents such as ethanol, hexane and ethyl acetate. 20 μg/mL of extracts were tested for their inhibitory effects on NO generation, NF- κB and β-secretase activities. Their anti-inflammatory effect was determined by NO assay in BV-2 cells, anti-amyloidogenic effect was determined by β-secretase activity assay in BV-2 cells as well as NF-κB luciferase activity in Raw 264.7 cells. MTT assay was also used to assess cell viability in neuronal cell. Those results are shown in Table 1. Overall, the anti-inflammatory and amyloidogenic activity of ethanol extract of *N. oceanica* were the most effective. The ethanol extract of *N. oceanica* decreased 37.15 % of NO level, 75.47 % of NF-κB luciferase activity and 14.04% of β-secretase activity induced by LPS.

**Table 1:** Inhibitory effect of extracts from marine microalgae on NO generation, NF-κB, BACE1 activity as well as cell viability

Species(extraction solvent)	NO inhibition(%)	NF-κBinhibition (%)	BACE1inhibition (%)	Cell viability (%)
*Isochrysis galbana* (EtOH)	34.79	35.60	No effect	91.58
*Isochrysis galbana* (hexane)	39.56	41.71	14.19	100.4
*Pavolva lutheri* (EtOH)	29.67	59.09	17.60	108.68
*Pavolva lutheri* (hexane)	39.58	78.62	21.11	76.16
*Arthospira plantensis* JD105 (EtOH)	35.89	47.67	10.05	83.5
*Arthospira plantensis* JD105 (hexane)	36.81	53.25	12.32	95.15
*Nanochloropsis oceanica* (EtOH)	37.15	75.47	14.04	139.44
*Tetraselmis suecica* (EtOH)	37.93	33.34	8.05	92.58
*Amphidinium carterae* (EtOH)	21.15	55.90	15.89	97.87
*Amphidinium carterae* (hexane)	42.82	29.95	0.57	95.16
*Amphidinium carterae* (ethyl acetate)	37.16	21.76	3.38	83.80
*Chaetoceros gracilis* (EtOH)	22.23	54.68	No effect	101.04
*Chaetoceros gracilis* (hexane)	30.86	20.97	1.42	102.63
*Chaetoceros gracilis* (ethyl acetate)	24.85	37.54	1.71	93.01
*Caetoceros difficuilis* (EtOH)	30.83	55.03	0.44	96.95
*Caetoceros difficuilis* (hexane)	32.28	41.97	11.61	106.88
*Caetoceros difficuilis* (ethyl acetate)	34.24	56.80	4.21	87.32
*Arthrospira platensis* (EtOH)	12.13	37.51	9.22	90.19
*Arthrospira platensis* (hexane)	19.21	39.99	16.31	115.23
*Arthrospira platensis* (ethyl acetate)	9.63	40.94	10.60	106.89
*Scenedesmus sp*. (EtOH)	16.86	12.09	No effect	94.76
*Scenedesmus sp*. (hexane)	23	8.49	No effect	115.24
*Scenedesmus sp*. (ethyl acetate)	12.63	26.67	22.32	78.70
*Nannochloropsis* (EtOH)	10.37	10.56	No effect	124.04
*Nannochloropsis* (hexane)	2.73	33.19	0.78	111.44
*Nannochloropsis* (ethyl acetate)	2.12	34.52	16.18	134.64
*Dunaliella saline* JD001 (EtOH)	16.59	31.91	11.19	118.86
*Dunaliella saline* JD001 (hexane)	9.18	65.33	16.48	128
*Dunaliella saline* JD001 (ethyl acetate)	34.79	66.06	33	138.07
*Achnanthidium sp*. (EtOH)	18.75	66.50	33.12	143.5
*Achnanthidium sp*. (hexane)	24.2	47.98	14.67	126.62
*Achnanthidium sp*. (ethyl acetate)	9.90	56.66	7.34	149.95

Different extracts of 32 marine microalgae were investigated for their anti-inflammatory effect using NO assay, anti-amyloidogenic effect using BACE1 activity assay in BV-2 cells and MTT assay in neuronal cells, and NF-κB luciferase activity in Raw 264.7 cell. The values are percentage of decreased activity.

### Effect of ethanol extract of *Nannochloropsis oceanica* on LPS-induced memory impairment

**Figure 1:**
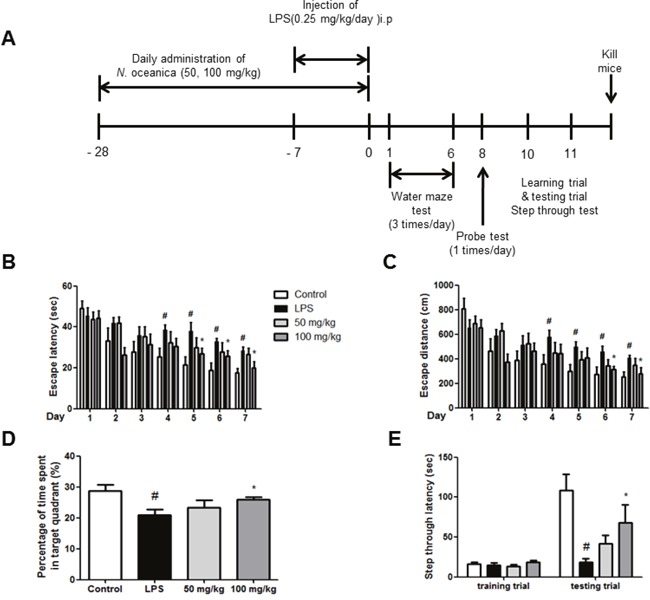
Effect of ethanol extract of *Nannochloropsis oceanica* on memory impairment Experimental scheme depicts about ethanol extract of *N. oceanica* treatment and LPS injection **(A)**. To investigate effect of ethanol extract of *N. oceanica* in LPS-induced memory impairment, we performed a water maze test **(B, C)**, probe test **(D)** and step-through type passive avoidance test **(E)**. Memory function was determined by the escape latencies (B, sec) and distance (C, cm) for 5 days, and time spent in target quadrant (D, %) in the probe test after administration of LPS. Each value is mean ± S.D. from 8 mice. *, Significantly different from control group (p < 0.05). #, Significantly different from LPS-treated group (p < 0.05).

To investigate the memory-improving effects of *N. oceanica* by ethanol extract on the LPS-induced memory impairment model, mice were continuously administered ethanol extract of *N. oceanica* (50, 100 mg/kg) in drinking water daily for 4 weeks (from day 1 to day 28), and then 250 μg/kg/day LPS was injected through i.p. daily for 1 week (from day 22 to day 28). All mice were trained for three trials per day for 7 days (Figure 1A). Escape latency and escape distances (Figure 1B, 1C) were determined for the effect of ethanol extract of *N. oceanica* on memory impairment. The average escape latency and swimming distance were about 17.5 ± 2.219 s and 253.2 ± 42.26 cm after 18 training trials in the control (saline) group. Average escape latency and swimming distance to the platform about 28.28 ± 1.958 s and 409.9 ± 19.75 cm in LPS-injected mice at day 7, while a significant decrease to 26.45 ± 3.174 s, 348.2 ± 57 cm in the 50 mg/kg group and 19.92 ± 3.073 s, 276.8 ± 49.51 cm in the 100 mg/kg group were observed in the LPS-injected mice that were given ethanol extract of *N.oceanica*.

The maintenance of memory function was tested with a probe trial one day after the water maze test. The average time spent in the target quadrant was decreased in the LPS-injected mice (20.86 ± 1.926%) compared to the control mice (28.67 ± 2.082%), but administration of ethanol extract of *N. oceanica* increased average time spent in the target quadrant to 23.35 ± 2.413% (50 mg/kg) and 25.92 ± 0.8397% (100 mg/kg) (Figure 1D). One day after the probe trial, a step-through test was performed. The control group exhibited an average step-through latency of 108.8 ± 19.96 s in the illuminated compartment, whereas that of the LPS-treated group decreased to 18.54 ± 4.305 s. The ethanol extract of *N.oceanica*-treated mice were recovered to 34.84 ± 9.06 s from the LPS-induced step-through latency (Figure 1E).

### Effect of the ethanol extract of *Nannochloropsis oceanica* on the activation of astrocytes and microglia in LPS-injected mice brain

**Figure 2:**
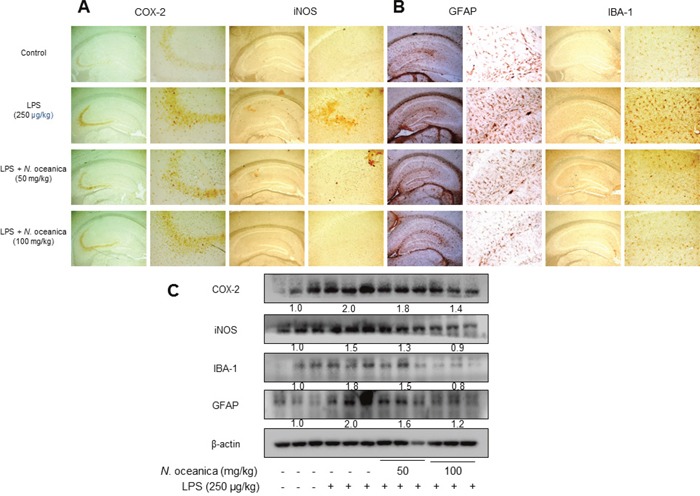
Effect of ethanol extract of *Nannochloropsis oceanica* on the LPS-induced neuroinflammation and amyloidogenesis in mice brain Immunostaining of COX-2, iNOS, GFAP and IBA-1 proteins in the hippocampus were performed in 20 μm-thick sections of mice brain with specific primary antibodies and the biotinylated secondary antibodies **(A, B)**. The expression of COX-2, iNOS, GFAP and IBA-1 were detected by Western blotting using specific antibodies in the mice brain. Each blot is representative of three experiments **(C)**. For the cropped images, samples were run in the same gels under same experimental conditions and processed in parallel. Each band is representative for three experiments.

It is well known that activated neuroglia increases amyloidogenesis and neuroinflammtion. The expression of inflammatory proteins (COX-2 and iNOS) was decreased with the treatment of ethanol extract of *N. oceanica* in the LPS-injected mice brain (Figure 2A). To see whether ethanol extract of *N. oceanica* could inactivate astrocytes and microglia, we performed an immunohistochemical analysis of GFAP (a marker of astrocyte activation) and IBA1-reactive cells (a marker of microglia activation) in the mice brains. GFAP- and IBA1-reactive cell numbers were significantly higher whereas the treatment of ethanol extract of *N. oceanica* reduced the number of GFAP reactive cells in the cortex and hippocampus of LPS injected mice brain (Figure 2B). Paralleled with the immunohistochemical results, Western blot analysis also showed that expression of COX-2 and iNOS as well as GFAP and IBA-1 was also significantly decreased in brain of ethanol extract of *N. oceanica* treated mice than LPS-injected mice brain (Figure 2C).

### Effect of ethanol extract of *Nannochloropsis oceanica* against the Aβ_1-42_ accumulation and amyloidogenesis as well as activation of NF-κB in LPS-injected mice brain

**Figure 3:**
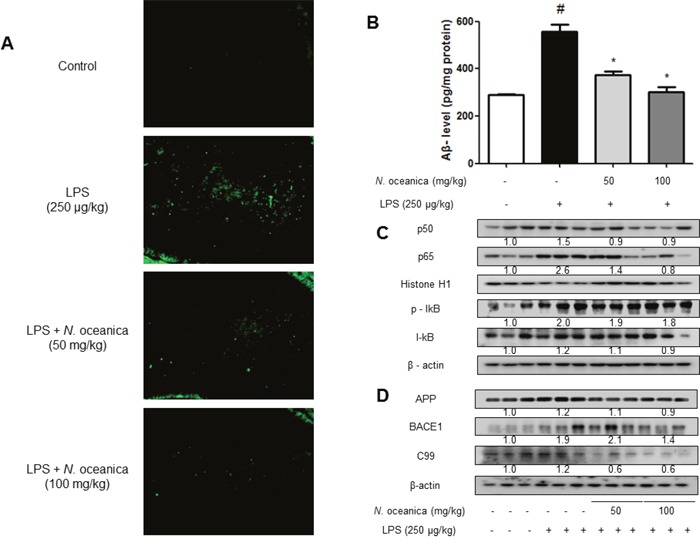
Effect of ethanol extract of *Nannochloropsis oceanica* on Aβ accumulation and expression of amyloidogenic proteins in the mouse brain Aβ accumulation in the brains of LPS-injected mice was determined by thioflavin S staining **(A)**. The level of Aβ_1-42_ was assessed by using a specific Aβ ELISA as described in Methods **(B)**. Phosphorylation of IκB, and p50 and p65 translocation were detected by Western blotting using specific antibodies in mice brain. β-actin and HistonH1 protein was used as an internal control **(C)**. The expression of APP, C99 and BACE1 was detected by Western blotting using specific antibodies in mice brain. β-actin protein was used as an internal control **(D)**. Each blot is representative for three experiments. Values measured from each group of mice were calibrated by amount of protein and expressed as mean ± S.D. (n = 8). *, Significantly different from control group (p < 0.05). #, Significantly different from LPS-treated group (p < 0.05).

Accumulation of Aβ could be associated with memory dysfunction. Thus, we determined the effect of ethanol extract of *N. oceanica* on the levels of Aβ in the brains of LPS-injected mice. Increased accumulation of Aβ was found in the brain of LPS-treated mice compared to non-treated mice brain. However, the accumulation of Aβ was inhibited by ethanol extract of *N. oceanica* treatment (Figure 3A). The ethanol extract of *N. oceanica* treatment also lowered the increased level of Aβ in LPS-injected mice brain (Figure 3B). NF-κB activity is implicated for amyloidogenesis and neuroinflammation. Thus, we determined NF-κB activation through the detection of p50, p65, and IκB phosphorylation. Phosphorylation of IκB and translocation of p50 and p65 were significantly decreased by the treatment of ethanol extract of *N. oceanica* (Figure 3C). We also investigated the levels of APP, BACE1 and C99 proteins using Western blot analysis. The expression of BACE1 and C99 was increased in the brains of LPS-injected mice, and this elevation was reduced by the treatment of ethanol extract of *N. oceanica* (Figure 3D). However, the expression of APP was not significantly changed.

**Figure 4:**
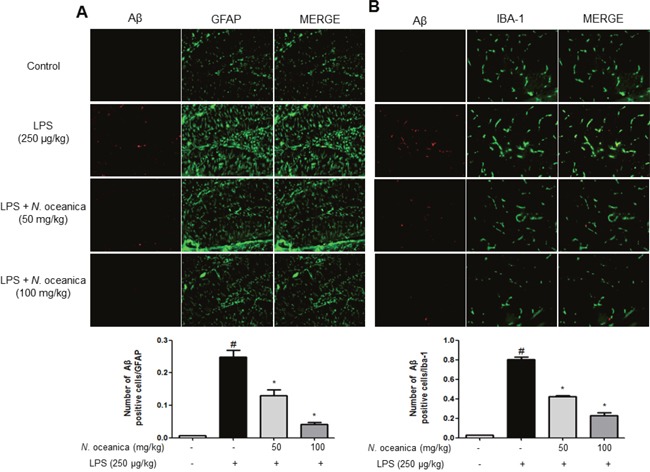
Effect of ethanol extract of *Nannochloropsis oceanica* on the LPS-induced neuroinflammation Staining was performed in 20 μm-thick sections of mice brain. Confocal microscope observation was performed as described in the Methods section. Immunostaining of GFAP (green) and Aβ_1−42_ (red) protein in the hippocampus was performed with specific primary antibodies, and fluorescence was developed using Alexa 488-conjugated anti-goat and Alexa 568-conjugated anti-rabbit secondary antibodies **(A)**. IBA-1 (red) and Aβ_1−42_ (green) protein in the hippocampus was performed with specific primary antibodies, and fluorescence was developed using Alexa 488-conjugated anti-mouse and Alexa 568-conjugated anti-rabbit secondary antibodies **(B)**. Similar patterns were observed in five mice brains.

The number of activated (GFAP-positive) astrocytes and accumulation of Aβ (Aβ-positive cells) were also determined by double immunofluorescence method. The co-reactive cell number for both markers was significantly increased by LPS injection, but was decreased by ethanol extract of *N. oceanica* treatment (Figure 4A). The co-reactive cell number for both activation of microglia (IBA1-postive cells) and Aβ accumulation (Aβ-positive cells) was also increased by LPS compared to the number in the non-treated mice brains, but was decreased by ethanol extract of *N. oceanica* treatment (Figure 4B).

### Ethanol extract of *Nannochloropsis oceanica* inhibits LPS-induced oxidative stress

**Figure 5:**
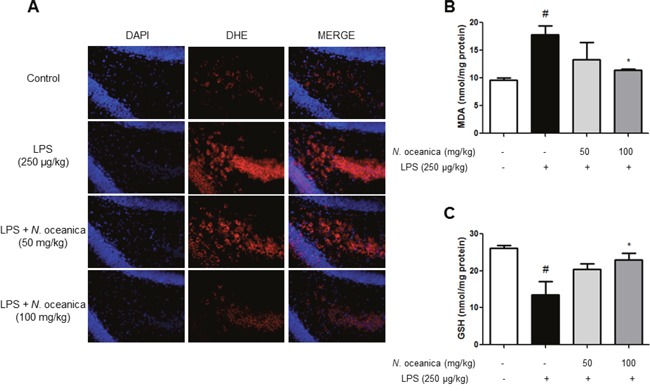
Effect of ethanol extract of *Nannochloropsis oceanica* on the LPS-induced oxidative stress in the mouse brain Intracellular superoxide radical production was measured by dihydroethidium in the brain. The brain sections were double stained with DHE (red) and DAPI staining (blue) **(A)**. MDA **(B)** and GSH level **(C)** were assessed by using a specific detection kit as described in Methods. Values measured from each group of mice were calibrated by amount of protein and expressed as mean ± S.D. (n = 8). *, Significantly different from control group (p < 0.05). #, Significantly different from LPS-treated group (p < 0.05).

Ethanol extract of *N. oceanica* decreased superoxide anion production in the mice brain. Intracellular superoxide radical production was measured by dihydroethidium [38] in the brain. Furthermore, another study has shown that local LPS administration contributes the activation of astroglial/microglial cells in the place of this toxin administration. Additionally, it was reported that the damage to the brain can be caused by inflammation and oxidative stress after longer exposure to LPS for 7 days or more [39, 40]. The accumulation of excessive intracellular ROS with increased enzymatic sources characterizes the oxidative stress [41]. Although the intensity of oxidative stress is different since oxidation usually occurs at a shorter time, systemic LPS treated for a long time will damage the brain with exposure to oxidative stress. The brain sections were double stained with DHE (red) and DAPI staining (blue). The ethanol extract of *N. oceanica*-treated mice had a significant decrease in the intensity of DHE signals compared to the LPS-injected mice (Figure 5A). We also evaluated MDA contents and GSH levels, which are indicators of oxidative stress. The MDA and GSH levels were significantly increased in the brains of LPS-injected mice compared to control mice. However, contrast to LPS-injected mice, mice treated with ethanol extract of *N. oceanica* showed lower MDA (Figure 5B) and GSH levels (Figure 5C).

## DISCUSSION

In this study, we found that ethanol extract of *N. oceanica* suppressed neuroinflammation, oxidative stress and amyloidogenesis in LPS-induced AD model, and thus ameliorated memory impairment. Growing epidemiological evidences have suggested that oxidative stress and neuroinflammation may contribute to the pathogenesis of AD [42, 43]. Recently, several researchers including ours reported that systemic administration of LPS induces the release of proinflammatory mediators and free radicals, and causes oxidative brain damage [27, 44]. Furthermore, systemic administration of LPS has been contributed to result in increased APP processing by β-secretase and intracellular accumulation of Aβ as well as cognitive impairment since LPS induces amyloidogenesis with concomitant increased neuroinflammation and oxidative damages [45, 46]. Furthermore, conventional transgenic models of AD are undoubtedly the basis of understanding several mechanisms in AD. However, the different method could be the bridge between AD and neuroinflammation since brain inflammation causes neurodegenerative diseases including AD. Moreover, administration of anti-inflammatory and anti-oxidative agents reduces the risk and delays the neuropathologic features of AD [47, 48]. In this present study, we reported for the first time so far that ethanol extract of *N. oceanica*, one of microalgae could decrease amyloidogenesis and memory deficiency via the prevention of oxidative brain damage and neuroinflammation.

The exact mechanism of amyloidogenesis is not clear. However, it is remarkable that NO and ROS have been implicated in the activation of BACE1 expression, where ROS has been hypothesized to increase β-secretase [49, 50]. In our previous study, L-theanine and EGCG which are antioxidant compounds showed anti-neuroinflammatory responses and anti-amyloidogenic activity through anti-oxidant mechanisms [51, 52]. Extensive evidence exists that ROS generation increases with Aβ as well as Aβ can also induce oxidative stress [53]. Alternatively, the inhibitory effect on NF- κB could be also associated with its anti-amyloidogenic effects. NF-κB is involved in the expression of inflammatory genes such as COX-2 and iNOS [54]. Moreover, the promoters of APP and BACE1 contain NF-κB sites, which derive transcription [35]. Some NSAIDs, including indomethacin and flurbiprofen have been shown to be effective at decreasing amyloidogenesis by targeting NF-κB [55, 56]. Additionally, numerous compounds which inhibit NF-κB were reported to attenuate amyloidogenesis such as sorafenib [57], artemisinin [58] and L-theanine [52]. We previously found that activation of NF-κB contributes to increasing β-secretase activity in neuronal cells expressing mutant PS2 [59], and also demonstrated that treatment of EGCG inhibits β-secretase activity via inhibition of NF-κB pathways in PS2 mice because it is a well-known anti-inflammatory and anti-oxidant agent [60]. In the present study, we found that ethanol extract of *N.oceanica* reduced ROS and MDA generation, and NF-κB activation. Thus, these antioxidative properties and inhibitory effect on NF-κB are associated with anti-amyloidogenic effects. Additionally, activation of astrocytes and microglia has been known to increase expression of BACE1, thereby increasing Aβ generation [61]. The increased expression of BACE1 after induction of chronic gliosis was not only associated with experimental mice brain but also in the brains of AD patients caused by Aβ plaques with activated microglia [62].

Thus, the present data indicated that the anti-inflammatory properties of ethanol extract of *N. oceanica* could also be associated with anti-amyloidogenesis through inactivation of microglia in the brain. Taken together, these data indicated that anti-oxidative effects, anti-neuroinflammatory effects and amyloidogenic effects of ethanol extract of *N. oceanica* could lead memory recovery. We recently found that the main component of *N.oceanica* is EPA, which has anti-oxidant and anti-inflammatory effects *in vitro* and *in vivo*. There are many sources of EPA, and the different composition of EPA could have an effect on differential pharmacological activities. Thus, we are studying the differential antioxidant and neuroinflammatory activities of EPA isolated from the ethanol extract of *N.oceanica*. An oral administration of 100 mg/kg/day for 4 weeks in our study did not cause any negative side effects. Thus ethanol extracts or its major component, EPA could be applicable for the development of functional food or drug for treatment of AD.

## MATERIALS AND METHODS

### Ethical approval

The experimental protocols were carried out according to the guidelines for animal experiments of the Faculty of Disease Animal Model Research Center, Korea Research Institute of Bioscience and Biotechnology (Daejeon, Korea) as well as Institutional Animal Care and Use Committee (IACUC) of Laboratory Animal Research Center at Chungbuk National University, Korea (CBNUA-929-16-01). All efforts were made to minimize animal suffering and to reduce the number of animals used. All mice were housed in a cage, three mice per cage, with automatic temperature control (21-25°C), relative humidity (45-65 %), and 12h light-dark cycle illuminating from 08:00 a.m. to 08:00 p.m. Food and water were available ad libitum. They were fed a pellet diet consisting of crude protein 20.5 %, crude fat 3.5 %, crude fiber 8.0 %, crude ash 8.0 %, calcium 0.5 %, phosphorus 0.5 % per 100 g of the diet (obtained from Daehan Biolink, Chungcheongbuk-do, Korea). During this study, all mice were specially observed for normal body posture, piloerection, ataxia, urination, etc. 2 times per day to minimize their pain and discomfort.

### Materials

The strains of all tested microalgae including *Nannochloropsis oceanica* were supplied from Natural Live Plankton (NLP, Busan, Republic of Korea) and identified by one of the authors (HSK)(Batch No. 285-F093). Grinded microalgae strain was disrupted by sonicator for 3 h. Then, finely powdered and dried microalgae were extracted with 100 % of EtOH, Hexane and Ethyl acetate one time (6 L × 1, 30 min) at room temperature by a funnel shaker. They were then passed to sedimentation tanks, which aim to remove the settleable solids by gravity. After sedimentation and filtration of extract, the collected filtrate, the extract solvent layer was dried overnight using a rotary vacuum evaporator and then, concentrated under reduced pressure. The main components in ethanol extract of *N. oceanica* are 12.8 % of docosapentaenoic acid (C22: 6, DHA), 5.3 % of eicosapentaenoic acid (C20: 5, EPA), 15.4 % of palmitic acid, 9.6 % of cholesterol, 11.9 % of palmitoleic acid, 4.5 % of 1-dodecanol 3, 7, 11-trimethyl-, 3.3 % of oleic acid chloride, and etc. The resulting products were further used for assay. Different extracts of 32 marine microalgae were investigated for their anti-inflammatory effect using NO assay, NF-κB luciferase activity and anti-amyloidogenic effect using BACE1 activity assay. The ethanol extract of *Nannochloropsis oceanica* was the most effective, so we administered ethanol extract of *N. oceanica* in *in vivo* experiment. The ethanol extract of *N. oceanica* (final concentration of 50 and 100 mg/mL) was dissolved in 100 % of dimethyl sulfoxide (DMSO), and stored at -20°C until use. The ethanol extract of *N. oceanica* (final concentration of 10 mg/mL) was dissolved in 100 % of DMSO, and aliquots were stored at -20°C until use in *in vivo*. The LPS was purchased from Sigma (serotype O55:B5, Sigma, St. Louis, MO. USA). The LPS (final concentration of 1 mg/mL) was dissolved in PBS, and aliquots in PBS were stored at -20°C until use.

### Animal experiment

Eight-to-ten week old male imprinting control region (ICR) mice (Daehan Biolink, Chungcheongbuk-do, Korea) were maintained and handled in accordance with the humane animal care and use guidelines of Korean FDA. ICR mice were randomly divided into four groups with 10 mice in each group: (I) Control group; (II) LPS group; (III) ethanol extract of *N. oceanica* (50 mg/kg) + LPS group control group; and (IV) ethanol extract of *N. oceanica* (100 mg/kg) + LPS group. The ethanol extract of *N. oceanica* was given to groups (III) and (IV) in drinking water daily for 4 weeks. Intraperitoneal (i.p.) injection of LPS (250 μg/kg) was administered to all groups except for the control group on the 4th week for 7 days. Control mice were given an equal volume of vehicle instead. The behavioral tests of learning and memory capacity were assessed using the water maze, probe and passive avoidance test. Mice were sacrificed after behavioral tests by CO_2_ asphyxiation.

### Morris water maze

A memory test was performed by the Morris's water maze test as described elsewhere with SMART-CS (Panlab, Barcelona, Spain) program and equipment [63].

### Probe test

Memory consolidation was tested with a probe test after 24h the water maze test with SMART-LD program (Panlab, Barcelona, Spain). Consolidated spatial memory was estimated by the time spent in the target quadrant area as described elsewhere [63].

### Passive avoidance test

The passive avoidance response was determined using a “step-through” apparatus (Med Associates, Georgia, VT) as described elsewhere [63].

### Microglial BV-2 cells culture

Microglial BV-2 cell cultures were prepared as previously described [64]. The cultured cells were treated simultaneously with LPS (1 μg/mL) and several concentrations (20 μg/mL) of marine microalgae were dissolved 100 % of DMSO. The cells were harvested after 24 h. NO level and β-secretase activity were determined.

### Neuronal cell culture

The Sprague-Dawley rats were maintained in accordance with the policy of the National Institute of Toxicological research, which is in accordance with the Korea Food and Drug Administration's guideline for the care and use of laboratory animals. Sprague-Dawley rats weighing 200-300 g were housed under 12 h light/dark cycles, at 23°C and 60 ± 5% humidity. All animals had free access to food (Samyang Foods, Seoul, Korea) and water. Cerebral cortical cells were isolated from neonatal rat brains (Day 1) in PBS (0.1 mol). Briefly, cerebral cortices were removed and incubated for 15 min in Ca2+- and Mg2+-free Hanks' balanced saline solution (Life Technologies) containing 0.2% trypsin. Cells were dissociated by trituration and plated into polyethyleneimine-coated plastic or glass-bottomed culture dishes containing minimum essential medium with Earle's salts supplemented with 10% heat-inactivated fetal bovine serum, 2 mM l-glutamine, 1 mM pyruvate, 20 mM KCl, 10 mM sodium bicarbonate, and 1 mM Hepes (pH 7.2). Following cell attachment (3-6 h after plating), the culture medium was replaced with a neurobasal medium containing B27 supplements (Life Technologies). The cells were cultured in the neuronal cell culture medium for 3 days, and then further cultured in a neuronal cell culture medium (NCM) with or without 20% astrocyte culture media (ACM). Experiments were performed with 4 to 6-day-old cultures; more than 90% of the cells in these cultures were neurons, and the remainder were astrocytes, as judged by cell morphology and by immunostaining with antibodies against neurofilaments and glial fibrillary acidic protein.

### RAW264.7 cell culture

The murine macrophage-like cell line RAW 264.7 was obtained from the American Type Culture Collection (Manassas, VA, USA), were cultured in Dulbecco's modified Eagle's medium (DMEM, Gibco-BRL) with 10% heat-inactivated fetal bovine serum (FBS) and penicillin/streptomycin (100 U/mL) at 37°C under humidified air contacting 5% CO_2_, inside a CO_2_ incubator as previously described [65].

### Brain collection and preservation

After behavioral tests, mice were perfused with phosphate-buffered saline (PBS) with heparin under inhaled CO^2^ anesthetization. The brains were immediately removed from the skulls and divided into left brain and right brain. One stored at -80°C, the other was fixed in 4 % paraformaldehyde for 72 h at 4°C and transferred to 30 % sucrose solutions, respectively.

### Immunohistochemical staining

Immunohistochemical staining was performed as described previously [66]. The sections were incubated overnight with a rabbit/mouse polyclonal antibody against GFAP; SC-33673 (1:300, Santa Cruz Biotechnology Inc. Santa Cruz, CA, USA), IBA-1; NB100-1028, iNOS; NB300-605 (1:300; Novus Biologicals, Inc., Littleton), COX-2; #12282 (1:300; Cell Signaling Technology, Inc., Beverly, MA). In order to prevent nonspecific staining, a blocking step was included. Sections were incubated at room temperature for 2 h with 5% bovine serum albumin [67] (in PBS), and then incubated overnight at 4°C with the primary antibody in blocking solution (5% BSA). Immunohistochemical staining was performed on 8 mice per group (3 sections per each mouse).

### Western blot analysis

Western blotting was performed as described previously [64]. To detect target proteins, specific antibodies against APP; NB110-55461, IBA-1; NB100-1028, iNOS; NB300-605 (1:1000, Novus Biologicals, Inc., Littleton), BACE1; #5606, COX-2; #12282 (1:1000, Cell Signaling Technology, Inc., Beverly, MA, USA), GFAP; SC-33673, p50; SC-114, p65; SC-8008, IκB; SC-371, phospho-IκB; SC-8404, β-actin; SC-47778, and Histone H1; SC-8030 (1:1000, Santa Cruz Biotechnology Inc. Santa Cruz, CA, USA) were used. The blots were then incubated with the corresponding conjugated goat anti-rabbit; SC-2004 or goat anti-mouse; SC-2005 or donkey anti-goat; SC-2020 IgG-horseradish peroxidase (HRP) (1:5000; Santa Cruz Biotechnology Inc. Santa Cruz, CA, USA) secondary antibodies. Immunoreactive proteins were detected with an enhanced chemiluminescence Western blotting detection system. The relative density of the protein bands was quantified with Image J software.

### Measurement of Aβ_1-42_

Lysates of brain tissue were obtained through a protein extraction buffer containing protease inhibitor. Aβ_1–42_ levels were determined using each specific ELISA Kit (CUSABIO) using a microplate absorbance reader (Sunrise^TM^, TECAN, Switzerland) after adding stop solution as described elsewhere [68].

### Thioflavin S staining

The thioflavin S staining was examined using a fluorescence microscope (Axio Observer A1, Carl Zeiss, Oberkochen, Germany) (×100) as described elsewhere [69].

### Fluorescence microscopy

The fixed cells and brain sections were exposed to the following primary antibodies: GFAP (1:100, Santa Cruz Biotechnology Inc. Santa Cruz, CA, USA), IBA-1 (Abcam, Inc., Cambridge, MA, USA), and Aβ (1:100, Cell Signaling Technology, Inc. Beverly, MA) at room temperature for 2 h. After incubation, the cells were washed twice with ice-cold PBS and incubated with an anti-rabbit or mouse or goat secondary antibody conjugated to Alexa Fluor 488 nm or 568 nm (Invitrogen-Molecular Probes, Carlsbad, CA) at room temperature for 1 h. Immunofluorescence images were acquired using an inverted fluorescent microscope Zeiss Axiovert 200 M (Carl Zeiss, Thornwood, NY) (×200).

### Nitric oxide determination

Cells were grown in 24-well plates and then incubated with or without LPS (1 μg/mL) in the absence or presence of various concentrations of microalgae for 24 h. The nitrite accumulation in the supernatant was assessed by NO detection kit (iNtRON, Kyungki-do, Korea). The absorbance at 520 nm was measured in a microplate absorbance reader, and a series of known concentrations of sodium nitrite was used as a standard.

### Oxidative stress assay

Hydrogen peroxides were measured according to the manufacturer's instructions (Cell Biolabs, San Diego, CA). Total glutathione (GSH), and malondialdehyde (MDA) were measured according to the manufacturer's instructions (Cayman chemical, USA). To perform assay, the brain tissues were homogenized, then normalized to protein concentration. Superoxide production in the brain was detected by dihydroethidium [38] staining (Sigma-Aldrich). Brains were incubated with 5 μM DHE for 30 min at 37°C in a humidified chamber protected from light. The average fluorescence intensity of the nuclei was then analyzed using Image Pro-Plus software (Media Cybernetics, Inc.).

### Assay of β-secretase activities

β-secretase activity in the mice brains was determined using a commercially available β-secretase activity kit (Abcam, Inc, Cambridge, MA, USA) using a fluorescence spectrometer (Gemini EM, Molecular Devices, California, USA) as described elsewhere [69].

### Reporter gene assay

Cells were plated at 1 × 10^5^ cells/well in a 24-well culture plate and transiently transfected with NF-κB-luciferase reporter (Affymetrix Inc., Santa Clara, CA, USA) or pNF-κB-luciferase reporter (Stratagene, Cedar Cree, CA, USA) using Lipofectamine LTX & PLUS (Invitrogen) in OPTI-MEM media (Invitrogen, Carlsbad, CA, USA) according to the manufacturer's instructions. The transfected cells were treated with LPS (1 μg/mL) in the absence or presence of various concentrations of *Nannochloropsis oceanica* for 24 h. The reporter gene activity was assayed using the luciferase assay kit (Promega Co., Madison, WI, USA), measured by a luminescence counter (Wallac Victor2 1420, PerkinElmer Inc., Waltham, MA, USA).

### Statistical analysis

All statistical analysis was performed with GraphPad Prism 5 software (Version 5.03; GraphPad software, Inc., San Diego, CA). Group differences were analyzed by two-way ANOVA followed by Dunnette's *post hoc* test. All values are presented as mean ± S.D. Significance was set at p < 0.05 for all tests.
